# Supplementary material for: Tumor-Intrinsic Enhancer of Zeste Homolog 2 Controls Immune Cell Infiltration, Tumor Growth, and Lung Metastasis in a Triple-Negative Breast Cancer Model
Source: Int J Mol Sci. 2024 May 15;25(10):5392. doi: 10.3390/ijms25105392 (PMC11121204; doi:10.3390/ijms25105392)
Supplement: Supplementary file 1 [file ijms-25-05392-s001.zip › ijms-2970566-supplementary.pdf]

TUMOR-INTRINSIC ENHANCER OF ZESTE HOMOLOG 2 CONTROLS IMMUNE CELL INFILTRATION, TUMOR GROWTH AND LUNG METASTASIS IN A TRIPLE-NEGATIVE BREAST CANCER MODEL

Lenore Monterroza,[1,2], Maria M. Parrilla[1], Sarah G. Samaranayake[3], Dormarie E. Rivera-Rodriguez[4], Sung Bo Yoon[5], Ramireddy Bommireddy[2], Justin Hosten[1], Luisa Cervantes Barragan[4], Adam Marcus[5], Brian S. Dobosh[1], Periasamy Selvaraj*[2], Rabindra Tirouvanziam*[1]

1. Department of Pediatrics, Emory University School of Medicine, Atlanta, GA 30322, USA.
2. Department of Pathology & Laboratory Medicine, Emory University School of Medicine, Atlanta, GA 30322, USA.
3. Emory University School of Medicine, Atlanta, GA 30322, USA.
4. Department of Hematology & Medical Oncology, Emory University School of Medicine, Atlanta, GA 30322, USA.
5. Department of Microbiology & Immunology, Emory University School of Medicine, Atlanta, GA 30322, USA

*Correspondence: tirouvanziam@emory.edu and pselvar@emory.edu

SUPPLEMENTARY MATERIAL

Supplementary Figure S1

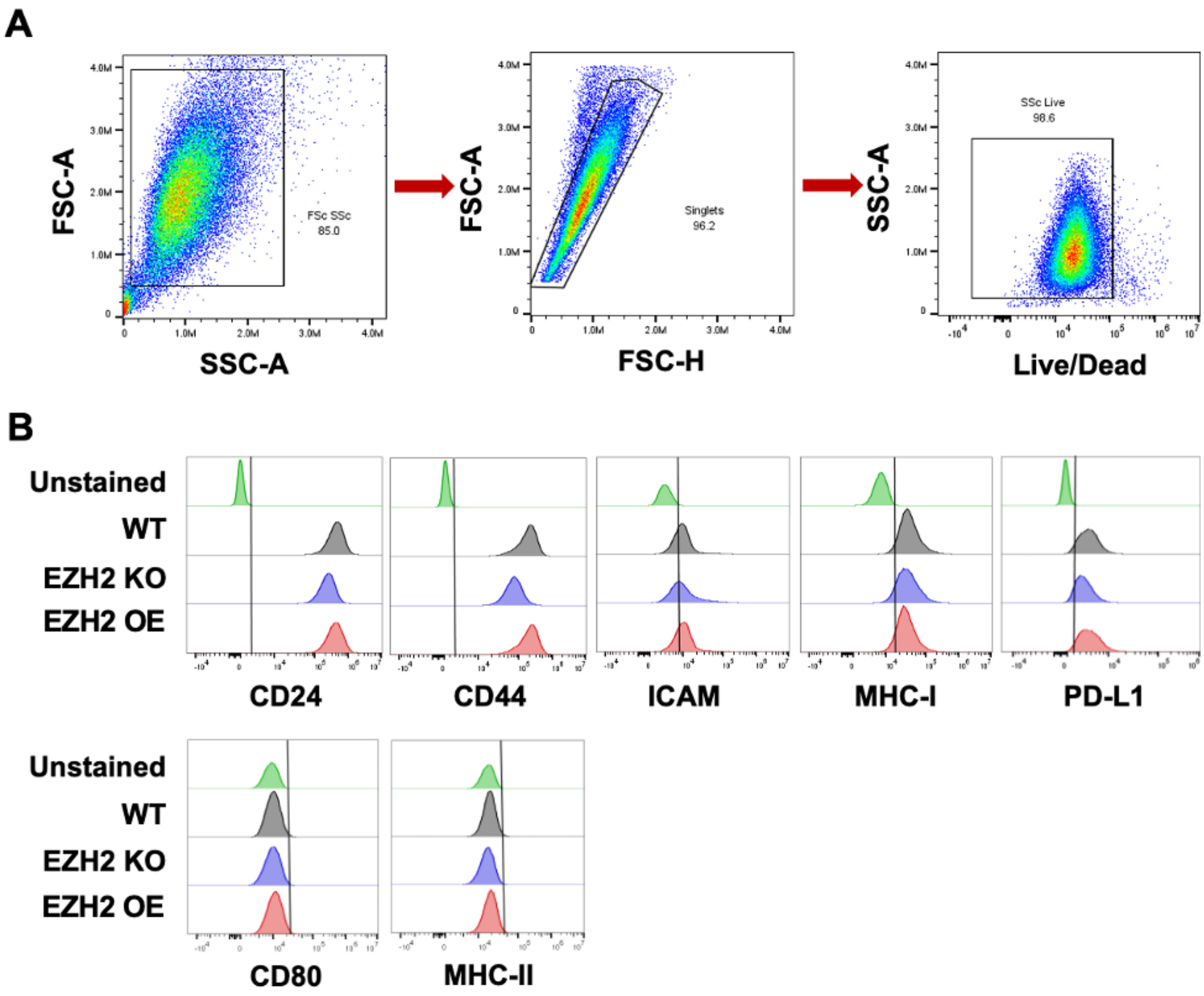

Figure S1. In vitro surface phenotype of EZH2 KO and EZH2 OE compared to parent WT 4T1 cells. (A) Gating strategy showing sequential cells, singlets and live gates. **(B)** Representative stacked histograms comparing expression levels of given phenotypic markers on stained EZH2 KO, EZH2 OE and WT 4T1 cell lines compared to unstained EZH2 KO cells (similar to EZH2 OE and WT 4T1 cells for baseline MFI in unstained samples). Dashed lines represent thresholds for marker positivity based on the upper boundary in respective unstained controls, revealing significantly expressed markers (top) and non-expressed markers (bottom).

Supplementary Figure S2

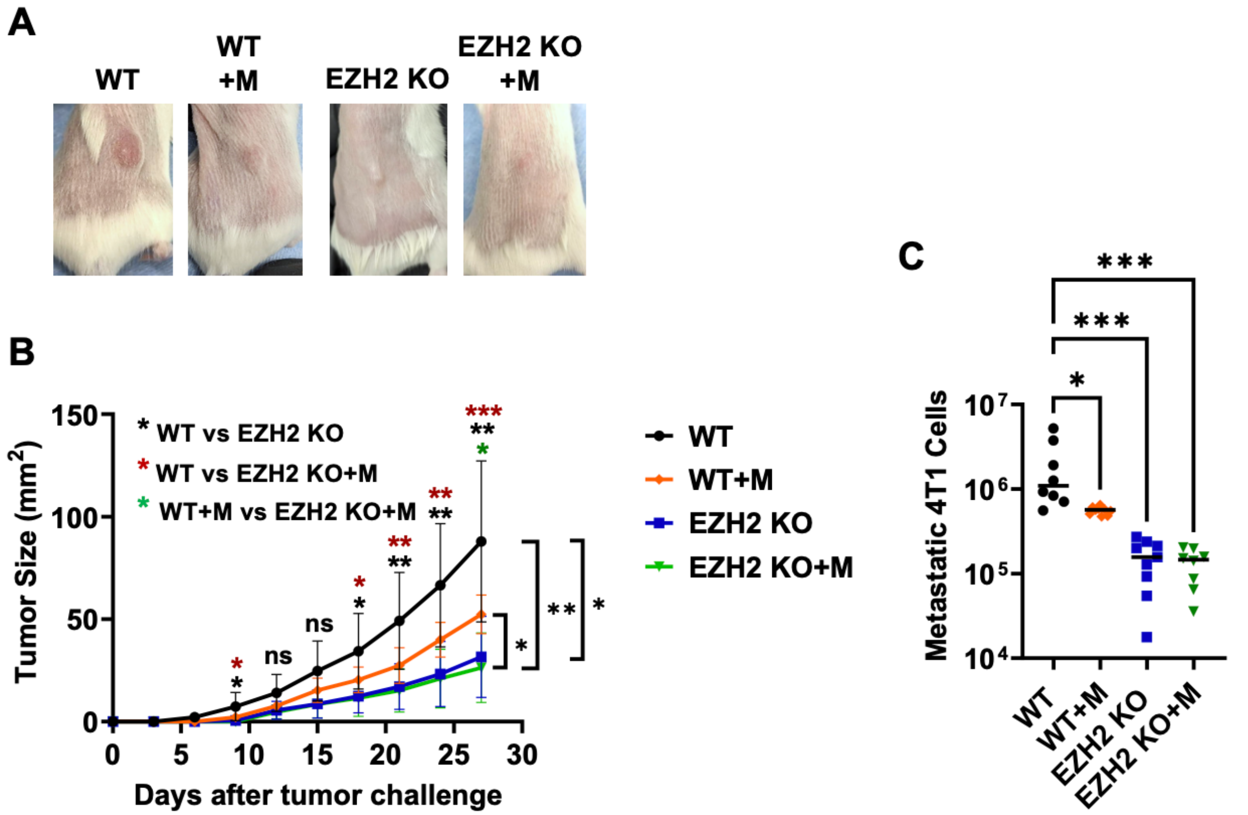

Figure S2. In vivo primary tumor growth and lung metastasis by EZH2 KO compared to parent WT 4T1 cells, in the absence or presence of STING agonist MSA-2. (A) Representative images of 4T1 WT and EZH2 KO primary tumors at day 18 post-injection are shown, grow either in the absence of MSA-2 (same as experiment presented in Figure 2), or in the presence of MSA-2 (+M). **(B)** Growth of 4T1 WT and EZH2 KO primary tumors over 28 days post-injection (n=6-7 mice per group, left), in the absence of MSA-2 (repeat of experiment conducted over 21 days, shown in Figure 2), or in the presence of MSA-2 (+M). Comparison across groups and timepoints is by two-way ANOVA with Tukey post-hoc test and shown as *$p<0.05$, **$p<0.01$, and ***$p<0.001$ (brackets). Comparison between groups at each timepoint is by one-way ANOVA and shown as *$p<0.05$ and **$p<0.01$ (as indicated for WT vs EZH2 KO, WT vs EZH2 KO+M, and WT+M vs EZH2 KO+M, above each timepoint). **(C)** Lung metastasis assay for 4T1 WT and EZH2 KO lines grown in the absence or presence of MSA-2 (+M). Comparison between groups is by one-way ANOVA with Tukey post-hoc test and shown as *$p<0.05$, and ***$p<0.001$.

Supplementary Figure S3

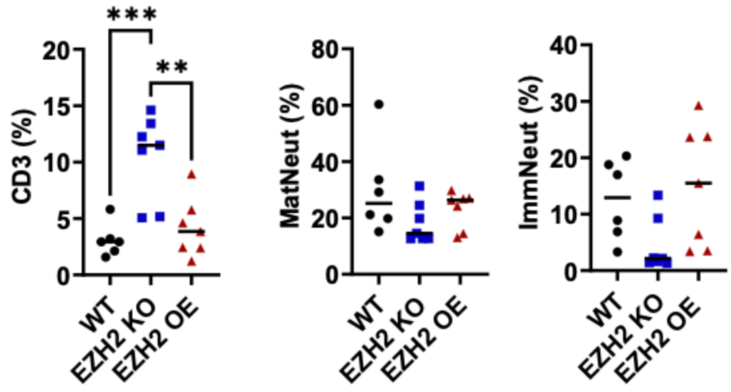

Figure S3. In vivo primary tumor infiltration by CD3+ T cells, mature and immature neutrophils for EZH2 KO and EZH2 OE compared to parent WT 4T1 cells. Relative percentages in 4T1 WT, EZH2 KO and EZH2 OE primary tumors of CD3+ T cells (left), mature neutrophils (middle) and immature neutrophils (right) gated among live leukocytes per **Figure 6A**. Comparison between groups is by one-way ANOVA with Tukey post-hoc test and shown as **$p<0.01$, and ***$p<0.001$.
